# A Pill of Laugh

**Published:** 1861-05

**Authors:** 


**A PILL OF LAUGH.**—The two favorite medicines with us are, an out-door walk or ride of two or three hours, and a good laugh. We are not exactly at leisure to hunt up fun, so we manufacture it for the nonce, or draw on the resources of memory. In other words, when we are sensible of the need of a stir-up, of a quickening of the fluids ethereal as well as material, we lie down on the floor, think of something funny and laugh at it; no grinning, but a real out-and-out, wide-mouth guffaw, which rings over the premises so loudly that the Quaker Upper House comes down to say, "William, I rather opine the neighbors will think thee a little daft;" these may not be the identical words, in fact, we rather think that a literal report would not "draw it quite so mild," no, not by a long shot; but the reader has the idea; now for the pills.

The two which are most infallible in their effects, and which do not seem to lose their power by repetition, are the reading of the "doubtful witness" in the January JOURNAL, to which we respectfully refer, price, ten cents; the other we have never seen in print, nor has any one else, perhaps from an impression it might be tabooed; but the incidents did occur, and the narration was made, and surely what has been stated in company may be put in print, especially as the distinguished conversationalist was a clergyman, a D.D., and belonged, too, to that most stern and staid and educated of all religions, the Old School Presbyterian Church; in short, no less a personage than the late Rev. Dr. , of , a man of infinite jest, who could tell a joke or take a glass of wine with any body. So sweet a voice had he, that it fell upon the ear like the most delightful "lullaby." Well, to our—pill of laugh. In one of his journeyings across the mountains to the "General Assembly" at Philadelphia, at that early day when it was made by "stage" instead of "rail," requiring weeks instead of days—the vehicle was full—a beautiful young country girl occupied the position of the end-seat of the middle bench, in the old-time famous "Troy coaches," hence she sat next the door, and it was most convenient that she should get in last. At one of the stopping-places for "refreshments," the horn summoned the "passengers" to resume their seats; the roads were muddy, the day was dismal, and the travelers were "dismaller," by reason of the fashion then, as now, to hurry you up before the dinner was half down. All were soon in but the doctor and the young lady. He opened the door, and was about handing her up to her seat, as any chivalric Kentuckian would do—in fact, they are all, that is, the race of forty years ago, as frank, and genial, and hospitable, and courteous, and brave a race of men as ever lived. Can't say as much of the later crops; rather think idleness and whisky, and the disposition to plume themselves on the virtues of their grand old sires have withered, and wilted, and wrecked the nobler nature, leaving behind a scaly race fit only to make loafers and braggadocios of. We are speaking of the sons only; the daughters! why, they are as handsome, and lady-like, and courteous, and courtly as they always were. Within a year the sweetest of them all said in our office: "Why, coz, when I returned to the old homestead to show my little boy to his grandfather, sixteen out of the seventeen young men with whom I had more or less an acquaintance, who were on visiting terms, have died drunkards or come to some other untimely end since I was a girl, a short seven years ago." In the same connection it may be related, that "the friend of our youth," from the same village school, and who has climbed higher up on the ladder of human fame and usefulness than we, passed a few days in our house not long ago, and in the mournfully pleasing reminiscences and inquiries which may be well imagined to have occurred on such an occasion, questions were asked of this and that and the other well-remembered name of childhood, he having come from the spot an age later than we. But rather objurgating as to the unsatisfactory answers given in reference to some of whom we wanted to hear, he explained: "The fact is, when I met my old friends, I was absolutely afraid to ask them about their children, 'gone to the dogs' being the very general and succinct history of those who, when I left a dozen or more years ago, were growing up as almost models of manliness and personal attractiveness. The reason was about the same as to all, idleness, gaming, drinking." Why, within a month when inquiring of an old acquaintance, of the young men who were in the medical department of the university at the time when a diploma in pills and physic was ground out to us, and who had then made themselves notable among their fellows by actual talents, and the promise of greater things to come—"Doctor, I

am ashamed to tell you, but in the town where I live, the place where you will always find the names 'at home' to which you have referred, is the billiard-saloon." Kentuckians! what means this? Do you intend that the race of giants shall die with Clay and Crittenden? Is it true, as reported in some of the papers, that your Governor was so "tight," on the occasion of a visit to the capital of the sister State of Ohio, that he denied it was three o'clock in the morning when he reached his lodgings, and as positive proof, said that it was only one o'clock, because it had that moment ceased, and it struck only one, for he had counted it three times? We have on several occasions boasted of the representation of our native county in this very same old Gotham, the clergy of several different denominations, the doctors of different names and pathies, and the editors! too, are literally headed by Bourbonians, the very identical place where the best whisky in the world comes from. Even the prince of horse-tamers, the immortal Rarey, got his cue from a man who was either a native of Bourbon county, or so near it that there was no fun in it. Dr. himself, the hero of this, what shall we call it? was bred in Bourbon, got his first fame and first wife there; but we forgot to remember two other celebrities from the same old "Bourbontown," as it was named seventy years ago. Both of them were our schoolmates; one is the prince of gamblers, and may be seen any day in Broadway "looking out" for spoils; the other was a prince of gin. Our most vivid recollection of the former was an effort of his, when not ten years old, to lead us into a "bet," which would have been inevitably lost by a quibble; this was seen instantly by us, and was declined. On a more recent occasion, the former being named "Asbury" by his good old Methodist mother, after her reverenced bishop, of blessed memory, the other was the son of a "new light preacher," of note in his day, "Augustus," these two worthies were in Broadway in broad daylight; they were two of the busiest men we ever saw, were Gust and Asbury. They were serious, too; there was not the scintillation of a smile. As we got nearer to them at the corner of Howard street and Broadway, there seemed to be an expression of countenance on both indicative of earnestness and determination; in short, they were endeavoring to hold up the lamp-post in that rather famous locality. Our impression was, as we passed rapidly by in a vehicle, that they would succeed; subsequent events verified that impression, for next day the lamp-post was there "all right," and no wonder, for they hugged it as a man would his own brother in affectionate circumstances.

Really, we think that the old United States had better not draft us to go down South on a fighting excursion, for if our leaden bullets were delivered as scatteringly as our pen missiles, we rather think the secessioners would not be much hurt. But as we were saying, when Dr.  was about handing up the unsophisticated "seventeen" to her seat, he found it occupied by a great, big, burly, frowslety-headed, unshorn, and rather garment-soiled new-comer who "didn't care whose seat it was; he found it empty and intended to keep it." This was one of those rather rare cases in which it was pretty clear that "parleying" would do no good, and the doctor both saw and felt it on the instant, and that the best thing to be done was a *coup de pied et de main;* so placing one foot on the stage-step as a *point d'appui*, or fulcrum, and one hand at the boor's throat, the "rough" was left sprawling in the mud; with the other hand he lifted the lass to her seat, and with the word "drive on!" to Jehu, the whip snapped, the horses snorted and away they went. The inmates were struck with silent surprise; for a few moments not a word was uttered; at length a fellow-traveler came to the relief of all—it was now quite dark—by remarking he had always heard the Kentuckians were afraid of nothing and nobody—in short, that they were a pretty fierce, ungovernable, dare-devil set. "You are entirely mistaken there," said Edgar; "when you get to know us better, you will find that we are an unforward, unpresuming, and in fact, rather a retiring and modest people, almost shame-faced sometimes; for example: A young clerical friend of mine had occasion to read the twenty-second chapter of Numbers before a large congregation. (The reader may remember that the chapter named, discourses somewhat about a remarkable mule, the head of which, or that of a number of its descendants, is either at Fowler's or Barnum's; Brady has the best photograph of them.) He found it necessary, when rising, to summon up his determination, and to assume a fearlessness which, in reality, he was conscious of not possessing. But on the faith of an axiom very true indeed in its immediate scope, that faint heart never won fair lady, he read ahead, with the same fearlessness that possesses a schoolboy, when he whistles with all his might in passing a graveyard. All went along splendidly until the close of the twenty-seventh verse. Then, there was evidently a hem and a hitch. I am only demonstrating to you, said the Doctor parenthetically, what a modest set we are out West. A kind of confusion seemed to come over the young man's eyes and mind; in short, there was such a mixture of horses and mules and other things, that the reader's eyes were all blurred over, and having some consciousness that attention was particularly upon him at that juncture, he made a desperate plunge, and suiting the action to the word, in a fairly thundering tone, he read, "And Balaam's HORSE opened at this juncture there was such an "irrepressible conflict" all over the house, that what the young man really said farther was never affirmed—it never "transpired." "Oh! no; you are mistaken," said the Doctor, "we are the most modestest people out West you ever saw."

